# Strategies to expand corporate autonomy by the tobacco, alcohol and sugar-sweetened beverage industry: a scoping review of reviews

**DOI:** 10.1186/s12992-022-00811-x

**Published:** 2022-02-14

**Authors:** Connie Hoe, Caitlin Weiger, Marela Kay R. Minosa, Fernanda Alonso, Adam D. Koon, Joanna E. Cohen

**Affiliations:** 1 Johns Hopkins Bloomberg School of Public Health, 615 N. Wolfe Street, Baltimore, MD 21205 USA; 2 Heidelberg Institute of Global Health, Faculty of Medicine and University Hospital, Heidelberg University, Im Neuenheimer Feld 130.3, 69120 Heidelberg, Germany

**Keywords:** Corporate strategies, Noncommunicable diseases, Commercial determinants of health, Tobacco industry, Alcohol industry, Sugar-sweetened beverage industry

## Abstract

**Background:**

Noncommunicable diseases contribute to over 70% of global deaths each year. Efforts to address this epidemic are complicated by the presence of powerful corporate actors. Despite this, few attempts have been made to synthesize existing evidence of the strategies used to advance corporate interests across industries. Given this, our study seeks to answer the questions: 1) Is there an emergent taxonomy of strategies used by the tobacco, alcohol and sugar-sweetened beverage (SSB) industries to expand corporate autonomy? 2) How are these strategies similar and how are they different?

**Methods:**

Under the guidance of a framework developed by Arksey and O’Malley, a scoping review was carried out whereby six databases were searched in June 2021 to identify relevant peer-reviewed literature. To be included in this review, studies had to explicitly discuss the strategies used by the tobacco, alcohol, and/or sugar-sweetened beverage multinational corporations and be considered review articles aimed to synthesize existing evidence from at least one of the three industries. Eight hundred and fifty-eight articles were selected for full review and 59 articles were retained for extraction, analysis, and categorization.

**Results:**

Results identified six key strategies the industries used: 1) influencing government policy making and implementation, 2) challenging unfavorable science, 3) creating a positive image, 4) manipulating markets, 5) mounting legal challenges, and 6) anticipating future scenarios. Despite these similarities, there are few but important differences. Under the strategy of influencing government policy making and implementation, for example, literature showed that the alcohol and SSB industries have been “privileged with high levels of participation” within international public health organizations.

**Conclusions:**

Understanding how industries resist efforts to control them is important for public health advocates working to reduce consumption of and death and diseases resulting from harmful commodities. Moreover, there is a greater need for the public health community to generate consensus about how to ethically engage or not engage with industries that produce unhealthy commodities. More studies are also needed to build the evidence base of industry tactics to resist regulation, particularly in the case of SSB, and in low-and middle-income countries.

**Supplementary Information:**

The online version contains supplementary material available at 10.1186/s12992-022-00811-x.

## Background

Commercial determinants of health (CDoH) have been defined as “strategies and approaches used by the private sector to promote products and choices that are detrimental to health” (p e.895) [1]. In an overview, Mialon (2020) [2] explained that CDoH includes three areas: 1) the unhealthy commodities that harm health; 2) the business, market, and political practices that harm health; and 3) global drivers, including market-driven economies that have facilitated these practices. In recent years, concerns about CDoH within the public health community have been augmented by the fact that these corporations are becoming increasingly more autonomous as a result of “1) rising demand, 2) increasing market coverage and 3) the continued internationalization of trade (p. e895)” [1]. The global expansion of corporate autonomy compromises government’s capacity to promote equality and health [3]. Corporate autonomy can be defined as the ability of a corporation to “establish its internal and external decision rules, its freedom to act according to its own rules, and its power to sanction non-conformist behaviour in its sphere of influence (p.9)” [4, 5]. In other words, it allows corporations the freedom to prioritize their interests at the expense of public interests, including public health [3]. While few private corporations enjoy full autonomy, corporations are persistent in their attempts to expand autonomy by preempting and resisting control [3]. Using Kickbusch et al’s [1] framework, corporate autonomy can be considered one of the drivers of ill-health influenced by and influencing the practices of corporations.

Noncommunicable diseases (NCDs) are a growing public health problem, resulting in over 70% of global deaths each year; the majority (85%) of deaths occur in low-and middle-income countries [6]. According to the World Health Organization, tobacco use, alcohol use, and consumption of unhealthy diets are key risk factors that contribute to two-thirds of NCDs [6]. While traditional approaches to addressing these so-called “lifestyle-related diseases” focused primarily on modifying individual-level behaviors, today, there is a growing recognition that key drivers of the NCD epidemic also include commercial determinants such as the policies and practices of transnational corporations that promote unhealthy commodities – namely the tobacco, alcohol and ultra-processed food and drinks industries [7]. Politically and economically influential, there is increasing evidence to show that these transnational corporations employ an array of tactics to influence the social and political environment, which then shapes the lifestyle and choice of consumers that ultimately influence health outcomes [1, 8–10].

Despite renewed attention, most of the existing work in this area has focused on the policies and practices of single industries that negatively impacts health [11]. While there are a few important exceptions [7, 12–15], limited studies have sought to systematically synthesize existing evidence across these three industries. In fact, to our knowledge only one framework has been developed to illustrate how corporations in general influence public health [8]. Existing studies that focused on more than one industry usually also highlight the similarities across these industries [7]. Further, researchers have highlighted the need to continue studying the various aspects of CDoH as drivers of ill-health and power [2].

In light of these gaps, the primary aims of this study are to answer the questions: 1) Is there an emergent taxonomy of strategies used by the tobacco, alcohol and sugar-sweetened beverage (SSB) industries to expand corporate autonomy? 2) How are these strategies similar and how are they different? By exploring patterns of corporate behavior, this research helps explain the social basis of corporate autonomy.

## Methods

A scoping review was carried out under the guidance of a framework developed by Arksey and O’Malley (2005) [16]; this approach was selected as we aimed to answer broad questions. The scoping review included the following steps: 1) identify the research question, 2) identify relevant studies, 3) study selection, 4) chart the data, 5) collate, summarize and report the results. A study team involving members with expertise in tobacco, alcohol and SSB was assembled.

The scoping review was guided by the following question (step one [16]), 1) Is there an emergent taxonomy of strategies used by the tobacco, alcohol and SSB industries to expand corporate autonomy? 2) How are these strategies similar and how are they different? For the purpose of this study, the industry is defined as multinational/transnational corporations involved in the production, distribution and marketing of tobacco, alcohol and/or SSB, as well as those supported by these corporations to maximize sales of their products. We define strategies broadly as corporate practices aimed at promoting corporate autonomy- a driver of ill-health; the term corporate practices is “the business and political activities of corporations. These practices result from companies’ decisions about the production, pricing, distribution, and promotion of their products and from their political efforts to create an environment favorable for their businesses (p. 87)” [17].

In consultation with a university librarian, C.W. organized our search terms around 4 concepts – (1) tobacco/alcohol/sugar-sweetened beverage, 2) corporation, 3) tactics, and 4) methodology: review/qualitative synthesis. For databases with indexing (e.g. Pubmed, Embase), the librarian advised the addition of additional relevant terms from the indexing list. Table 1 illustrates an example of our search strategy.

**Table 1:** An example of our search strategy

Database	Concept	Search terms
Pubmed *N* = 1314	1) tobacco, alcohol, sugar-sweetened beverage	“tobacco use”[mesh] OR “Tobacco Industry”[mesh] OR “Tobacco Products”[mesh] OR “Tobacco Smoking”[mesh] OR “Electronic Nicotine Delivery Systems”[Mesh] OR “vaping”[Mesh] OR “vaping”[tw] OR “tobacco product*”[tw] OR “cigar*”[tw] OR “bidi*”[tw] OR “pipe tobacco*”[tw] OR “tobacco use”[tw] OR “smokeless tobacco*” OR “tobacco industry”[tw] OR “Electronic Nicotine Delivery Systems”[tw] OR “alcohol industry”[tw] OR “Alcoholic Beverages”[mesh] OR “beer”[mesh] OR “wine”[mesh] OR “Alcoholic Beverage*”[tw] OR “liquor*”[tw] OR “beer”[tw] OR “wine”[tw] OR “Sugar-Sweetened Beverages”[mesh] OR “Artificially Sweetened Beverages”[mesh] OR “Sugar Sweetened Beverage*”[tw] OR “Sugar Added Beverage*”[tw] OR “Sugar Sweetened Soda*”[tw] OR “Sweetened Beverage*”[tw] OR “diet soda*”[tw] OR “diet beverage*”[tw] OR “soft drink*”[tw] OR “beverage industry”[tw]
	2) corporation	“Corporate”[tw] OR “industry”[tw] OR “company”[tw] OR “companies”[tw] OR “business”[tw] OR “firm”[tw] OR “firms”[tw]
	3) tactics	“Commerce”[Mesh:NoExp] OR “Marketing”[Mesh:NoExp] OR “Advertising”[mesh] OR “Direct-to-Consumer Advertising”[mesh] OR “advertising*”[tw] OR “marketing”[tw] OR “Direct to Consumer Marketing*”[tw] OR “tactics”[tw] OR “tactic”[tw] OR “strateg*”[tw] OR “interference*”[tw] OR “lobbying”[mesh] OR “lobby*”[tw] OR “public opinion”[tw] OR “polic*”[tw] OR “influence”[tw] OR “corrupt*”[tw]
	4) review/qualitative synthesis	“Review” [Publication Type] OR “review”[tw] OR “qualitative synthesis”[tw] OR “qualitative evidence synthesis”[tw] OR “industry document*”[tw] OR “Documentation*”[Mesh]

C.W. carried out the search in six different databases (PubMed, Scopus, Embase, Web of Science, Business Source Ulimate, and EconLit) in June 2021. Covidence (https://www.covidence.org/home) was used to manage study selection and evaluation. A total of 10,198 articles were identified, of which, 3962 articles were duplicates and removed. All articles were first screened based on title and abstract by C.W., C.H., and M.M based on the following inclusion criteria:

- Be published in peer-reviewed journals from 1984 to June 2021;
- Be published in English;
  Be a review article aimed at synthesizing existing evidence from at least one of the three industries. Under the guidance of the typology of reviews developed by Grant & Booth’s [18], reviews were included if the authors indicated a systematic approach in their peer-reviewed literature searches (e.g. systematic reviews, scoping reviews, realist reviews, qualitative evidence synthesis, etc.). We also included realist and rapid reviews that were not mentioned in Grant & Booth’s article; and
- Discussed the strategies used by tobacco, alcohol, or SSB multinational/transnational industries (note: if the article discussed food and beverage industry, we read them in full, and included these manuscripts if they mentioned SSB industry specifically).

**Fig. 1:**
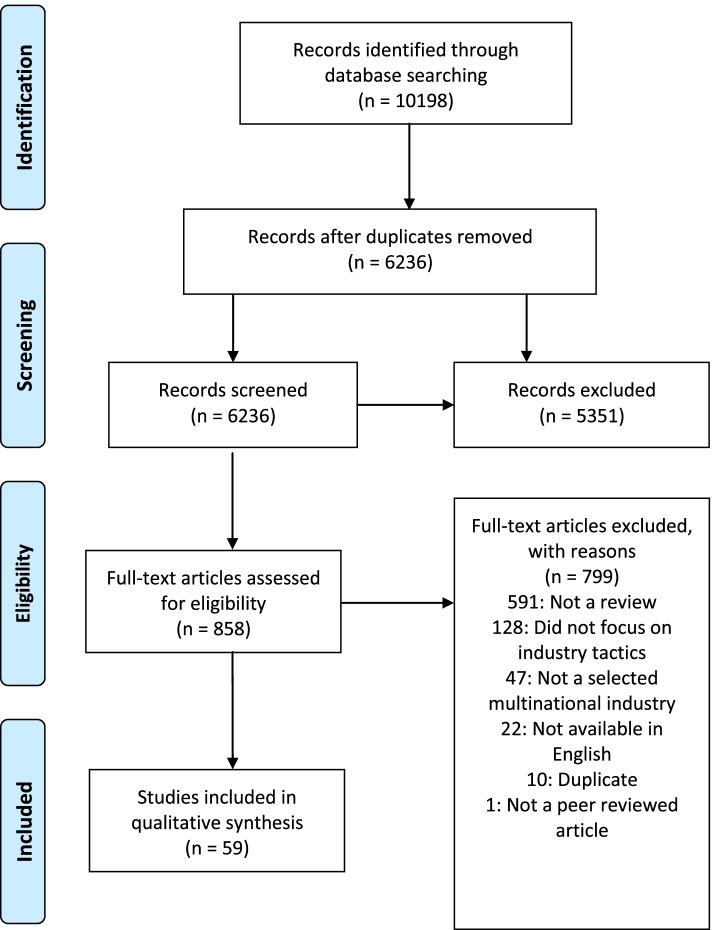
PRISMA flowchart

If inclusion criteria were met, articles were forwarded to full-text review (*n* = 858) (step 2 [16]). Articles were excluded if they were 1) not published in peer-reviewed journals, 2) were not published in English, 3) did not use a systematic approach to their literature search, 4) did not contain peer reviewed literature in their review, and 5) did not discuss the strategies used by at least one of the three multinational/transnational industries. Questions regarding inclusion were discussed among M.M., C.W., F.A., and C.H. during regular meetings. Fifty-nine articles were retained for extraction, analysis, and categorization (step 3 [16]). See Fig. 1 for an overview of the review process.

### Analysis

The charting process (step 4 [16]) included full text review where key themes and quotes exemplifying each tactic mentioned were extracted from each articles. C.W., C.H., M.M. and F.A. charted the articles. A preliminary taxonomy of strategies was developed by the Johns Hopkins Institute for Global Tobacco Control through group discussions, existing frameworks on tobacco industry tactics, and watchdog websites to identify tobacco industry tactics prior to this study. Five tactic domains were established: mounting legal challenges, influencing government policymaking and implementation, manipulating markets, creating a positive public image, and challenging science. Authors used this initial taxonomy as a starting point for charting included articles. We started with this approach because more studies have been carried out in the area of tobacco industry interference.

The collation, summarization, and reporting of findings (step 5 [16]) was overseen by C.H. Deductive and inductive coding were used to allow for new themes to emerge. Emerging themes were discussed between authors involved in the charting process. Theme names as well as categorization of subthemes within themes and consolidation of subthemes were discussed. C.H. reviewed all themes, subthemes, and categorization of article quotes within these categories to ensure that themes/subthemes were being consistently assigned and sufficient detail was captured to categorize content into themes. Collated articles were characterized by industry and strategies [Supplement 1 Extraction Table]. Gaps in research between industries were identified, as well as similarities and differences between industry strategies. We also selected quotes that best exemplified the selected tactics to be included in the results section.

## Results

We observed broad trends in the published literature on corporate strategy in tobacco, alcohol, and SSB industries. First, about 50% of the reviews were published between 2019 and 2021, suggesting that this is a growing area of research. Second, 47% of all reviews focused specifically on the tobacco industry, 24% on the alcohol industry, 17% on the SSB industry, and 12% on more than one industries. This trend appears to be changing, particularly since 60% of the SSB articles were published between 2020 and 2021. Similarly, multi-industry studies are becoming more common, with six out of the seven multi-industry reviews published between 2020 and 2021.

The majority of the reviews were systematic reviews (36%), followed by literature reviews (31%), and narrative reviews (14%). About 5% were qualitative syntheses, 5% were others (e.g. rapid review, overview with methods), 3% were scoping reviews, 3% were realist reviews, and 3% were mixed methods reviews.

Table 2 summarizes a taxonomy of strategies industry actors use to expand corporate autonomy: 1) influencing government policy making and implementation, 2) challenging unfavorable science, 3) creating a positive image, 4) manipulating markets, 5) mounting legal challenges, and 6) anticipating future scenarios. Multi-industry reviews included findings from at least two of the three industries.

**Table 2:** Taxonomy of Strategies used by the Tobacco, Alcohol and SSB Industries

	Tobacco (***N*** = 28)	Alcohol (***N*** = 14)	SSBs (***N*** = 10)	Multi-industry (***N*** = 7)
**Influencing government policy making and implementation**				
Lobbying	13	6	3	5
Revolving door	1	1	1	0
Policy capture	1	2	0	2
Intimidation, incentives & bribery	7	4	2	2
Developing/promoting alternative solutions	2	2	0	0
Influencing voters and the general public	7	1	0	1
**Challenging Unfavorable Science**				
Shaping the evidence-base	12	7	4	1
Infiltrate the public health scientific community	1	1	0	0
Hiding industry role in research	2	1	0	0
**Creating a positive image**				
Corporate social responsibility	8	7	4	3
Offering voluntary self-regulation	3	4	1	1
Issue framing	1	3	1	1
Targeted marketing	6	3	1	2
Creating “healthier” products	3	0	0	1
**Manipulating Markets**				
Manipulating cost	3	0	3	1
Economic framings	7	0	0	2
Undermining tax policies	2	0	1	1
Illicit trade and smuggling	6	1	0	1
**Mounting Legal Challenges**				
Litigation	8	1	1	1
Using trade agreements/investment law to challenge national policies	2	0	1	1
Trade Framing	0	0	0	1
Preemption	2	0	0	0
**Anticipating future scenarios**				
Expanding into emerging markets	2	0	2	1
Monitoring and discrediting public health proponents	2	0	1	0

### Influencing government policymaking and implementation

Available evidence showed that tobacco, alcohol, and SSB industries all seek to influence government policymaking and implementation to expand corporate autonomy. They do so by engaging in six key activities (Table 1). The first, and by far the most prominent in the literature, is *lobbying*. There were different forms of lobbying found across the three industries: lobbying directly or through the use of third parties [19–42], coalition and constituency building [26, 28, 31, 32, 36, 38–40, 42, 43], and the use of public-private partnerships [31, 32, 39, 44, 45]. In the last case, we found that alcohol and SSB industries have also been “privileged with high levels of participation” within international public health organizations [24].

Tied to lobbying is the use of the *revolving door*, referring to the back-and-forth movement of individuals between roles as legislators and regulators and members of the industries affected by legislation and regulation. This conflict of interest was seen across all three industries [31, 40, 41]:

*“After the experience with the SSB tax, the National Association of Beverage Producers became A.B. Chile, and hired a former member of parliament and prominent politician to be its representative”* [41].

The third activity used by these industries to influence government policymaking and implementation is *policy capture* [2, 24, 28, 46, 47]. In these instances, industries capture different branches of government to instigate conflict between ministries [2, 28] and/or directly help governments draft legislation [24, 47]:

*“A 2009 analysis of draft alcohol policy texts in Uganda, Malawi, Lesotho and Botswana e.g. found that as a result of significant industry input, alcohol policies in all four countries largely reflected industry interests“* [24].

Fourth, there was evidence of *using intimidation, incentives and bribery* across all three industries [22, 25–27, 30, 31, 35, 36, 38–40, 43, 46, 48]. This was done either through direct donations or gifts to politicians, policy makers or political parties [22, 25, 30, 35, 39, 40, 43] to support industry positions or funding of alliances, public health organizations, and front groups [27, 39, 46, 48]. The Mexican Federation of Diabetes and Funsald, for example, ceased advocating for health system reform after receiving funding from Coca-Cola [48].

Fifth, all three industry actors work to *influence voters and the general public*, often through the strategic use of media [26, 28, 30–32, 49, 50]. The tobacco industry, for example, paid for advertisements to drum up support for the industry position. These advertisements spread the idea that the policy proposed would be harmful to small businesses and farmers [49].

Finally, alcohol and tobacco industry actors also *develop and promote alternative solutions*, like non-regulatory initiatives with limited or no effectiveness [31, 32, 42, 45]. One study showed that the alcohol industry distanced itself from the tobacco industry when being involved in activities aimed at influencing policies [33].

### Challenging unfavorable science

Studies showed that tobacco, alcohol, and SSB industry actors challenge science *by shaping the evidence base* through funding research that supports industry favorable results [19, 26, 35, 36, 38, 49, 51–59].

*“Studies funded by the SSB industry were significantly more likely to find no associations (between SSB and obesity/diabetes) than independently funded ones”* [57].

These research studies are funded directly from the industry and, at times, indirectly through third parties to create distance from corporate interests [27, 53]. The alcohol industry-funded International Center for Alcohol Policies (ICAP), for example, has been involved in an array of research activities in low-and middle-income countries [27, 53]. Available evidence also revealed that these industry actors *shape the evidence base* through recruiting and co-opting researchers/scientists and/or physicians [19, 26, 28, 53, 60], commissioning, writing, and promoting industry favorable research and reports [27, 31, 32, 42, 52], attacking the source of public health information, [28, 32, 36, 53] misrepresenting existing evidence [32, 53]*,* and conducting research itself [40].

*“An ARA-commissioned report concluded that: (a) advertising is not linked to consumption, (b) advertising bans would be ineffective, (c) the alcohol industry contributes much to South Africa’s economy and (d) alcohol-related problems are primarily the fault of the informal market”* [42].

Several articles also highlighted how tobacco and alcohol industry actors have tried to *hide its involvement in research;* specifically, the funding of research [19, 61]:

*“To slow acceptance of the scientific evidence on the dangers of SHS [secondhand smoke] that were driving legislation and regulation around the world, the TTCs [transnational tobacco companies] secretly recruited scientists and physicians from around the world and funded research without publicly disclosing the source of funding to counter scientific claims regarding SHS”* [19]*.*

In addition, tobacco and alcohol industry actors have also *attempted to infiltrate the public health scientific community* by developing close relationships with scientific bodies and attending/sponsoring conferences [26, 53].

### Creating a positive image

Tobacco, alcohol, and SSB industry actors all seek to create a positive image of themselves and their products by engaging in three key activities (Table 1). First, through *corporate social responsibility (CSR) activities* these industry actors portray themselves as good corporate citizens who are part of the solution [19, 21, 26–29, 32, 34, 36–38, 40, 41, 43–45, 51, 52, 59–63]. Philip Morris, for example, started “Good Agricultural Practices” [61], Diageo’s Responsible Drinking Fund supported prevention programs in over 40 countries [32], and Coca-Cola sponsored more than 150 physical activity programs in more than 100 countries through its “Active Health Living” program [62]. Existing studies showed that CSR also helped enhance the credibility of industry actors [21, 34] and has been used for economic, political and, marketing purposes [27, 29, 63].

*“ The alcohol industry’s“corporate social responsibility” campaigns have been more effective at improving brand loyalty and industry reputation than achieving public health outcomes (Mart, 2013). These campaigns strengthen the industry’s commercial interests, while failing to reduce harmful alcohol use (Babor et al., 2018)”* [51].

CSR also lends itself well for industries to argue in favor of self-regulation, thus expanding corporate autonomy. Studies showed that all three industry actors *have offered voluntary self-regulations* [19, 26, 31, 32, 36, 42–44, 59, 64].

*“Industry representatives have reiterated their commitment to combating obesity through measures including sponsoring children’s sporting programs, providing nutrition and physical activity information and implementing voluntary self-regulatory codes on advertising to children. These initiatives, considered acts of corporate social responsibility, are designed to build a positive brand image and to counter public criticisms of the industry and its contribution to poor health”* [43].

Third, industry actors engaged in *issue framing* [27, 28, 33, 34, 39, 40]. Existing studies show that all three industries try to frame their issue as a matter of personal choice and individual responsibility [27, 34, 40]. The alcohol industry actors, for example, portray the issue of excessive drinking as one that is limited to an aberrant minority (e.g. binge drinkers) rather than a widespread population-level problem [40]. Such portrayal also allow industry actors to make arguments against population-level policies [30].

Fourth, studies showed that the tobacco, alcohol, and SSB industries engage in targeted *marketing*, including to youth, women, and racial and ethnic minority groups [19, 20, 22, 26, 37, 39, 42, 47, 65–68]. One of the reasons for the use of this tactic is to boost the reputation of the industry:

“Several analyses describe the tobacco industry’s history of targeting AAs [African Americans] with menthol cigarette marketing and donations to AA leadership organizations to improve its reputation in these communities” [65].

Finally, industry actors also create greater variation of products to maintain existing users, attract new users, and, in some circumstances, to demonstrate good corporate citizenship [19, 21, 26, 69, 70]. This includes the creation of “healthier” or “reduced harm” products and engaging in product reformulation, which has been described as a “regulation avoidance tactic” [17].

### Manipulating markets

Tobacco and SSB industry actors *manipulate cost* through an array of pricing strategies such as engaging in price discrimination and price promotions [36, 39, 50, 71–74]. These actors also use economic framings to [24, 26, 28, 30, 31, 35–37, 48] for example, *highlight their economic importance* to the country in order to fight against the adoption and implementation of public health policies [24, 26, 35–37, 48].

*“STMA/CNTC [State Tobacco Monopoly Administration/China National Tobacco Corporation] also interferes with the political and legislative processes of tobacco control by exaggerating the economic importance of the industry”* [48]*.*

Tobacco and SSB industry actors also *undermine tax policies* by under shifting (absorb tax increase), over shifting (increase retail price by an amount greater than the tax), and influencing tax structures [36, 43, 52, 72].

*“This indicates that tobacco companies have absorbed the tax adjustment, bearing the burden of the increased tax. The increased tax rate has little impact on the retail price of cigarettes and thus has no impact on consumers”* [52].

Available studies also showed that tobacco industry actors are involved in *illicit trade and smuggling* [19, 20, 26, 36, 37, 75] and tobacco and alcohol industry actors use the potential for an increase in illicit trade, smuggling and black markets respectively as arguments against public health policies that would reduce product demand [26, 30, 47].

### Mounting legal challenges

Legal challenges have also been used by all three industries to expand corporate autonomy. Legal challenges can be divided into four main activities: *litigation*, *using trade agreements/investment law to challenge national policies*, *trade framing*, and *preemption*. When looking at the different examples of how these industries undertake litigation in the literature, we found the use and/or threat of *litigation*, where the industries threaten to sue in order to frighten and overwhelm opponents into opening up new markets, to undermine national health policy and/or to challenge international law [20, 26, 28, 30–32, 35, 36, 38, 41, 75].

*“Since the implementation of the law [SSB tax law], TNCs [transnational corporations] have filed several lawsuits against the Chilean State challenging the legality of restricting their trademarks, cases which are still pending”* [41].

Using trade agreements/investment law to challenge national policies was also captured in reviews of all three industries [24, 37, 41, 45].

*“While THCCs [transnational health-harmful commodity corporations] cannot themselves bring claims against governments at WTO [World Trade Organization] for violating international trade obligations, there is evidence that corporations use international trade-related legal threats in an attempt to force involuntary public health policy non- decisions and prevent policy transfer regionally or globally, especially for tobacco control”* [24].

Likewise, available evidence also showed that the tobacco industry used economically vulnerable low-and middle income countries to serve their interest. These LMICs, for example fought against Australia’s plain packaging law as well as Canada’s tobacco additives ban in WTO forums with the use of *trade arguments* [24]. Such arguments were also used to water down Colombia’s alcohol health warning label law [24].

In addition to the aforementioned tactics, the tobacco industry used *preemption* of local tobacco regulation to undermine public health [25, 31]. Preemption occurs when a higher level of government limits or eliminate the authority of lower levels of government to enact stronger laws [76].

### Anticipating future scenarios

Tobacco, alcohol, and SSB industries *anticipate future scenarios*. In recent years, these industry actors have been *expanding into emerging markets* to counteract declining sales in high-income countries [2, 19, 26, 39, 74]. Available evidence, for example, showed that tobacco companies like British American Tobacco, Imperial Tobacco, Philip Morris International, and Japan Tobacco International started targeting Southeast Asian markets soon after tobacco consumption began to decline in high-income countries [26].

Studies also showed that tobacco and SSB industry actors *monitor and discredit public health proponents* in order to devise counter strategies in anticipation of future scenarios that jeopardizes corporate autonomy [26, 28, 55]. One article described how tobacco companies “create tensions between LDCs [less-developed countries] and OECD countries and between public health [i.e. communicable diseases] and environment [including non-communicable diseases]” [28].

## Discussion

Our study identified six main strategies used by the tobacco, alcohol and SSB industry actors to expand corporate autonomy: 1) influencing government policy making and implementation, 2) challenging unfavorable science, 3) creating a positive image, 4) manipulating markets, 5) mounting legal challenges, and 6) anticipating future scenarios. These findings support existing work that have underscored how unhealthy commodities industries utilize a similar playbook to undermine public health [7]. Results also align well with existing frameworks including the five vehicles of power described by Lima & Galea (Political Environment, Preference Shaping, Knowledge Environment, Legal Environment, and Extra-Legal Environment) [8]. Our study, however, sheds light on two additional industry tactics – 1) manipulating markets and 2) anticipating future scenarios- that were not captured by the framework.

A key reasons for the overlap in strategies is the various ways in which these industries are linked [77, 78]. Studies, for example, have shown that tobacco and alcohol companies forge direct partnerships, as well as share information and strategies [79–81]. Internal tobacco industry documents have also revealed that many SSB companies were previously owned by tobacco companies; three of the top 10 SSB companies in Asia-Pacific also produce alcohol products [82]. Further, some third party facilitators, such as public relations firms, that have worked with the tobacco industry have also been employed by other industries. Likewise, marketing firms have supported coordinated campaigns between tobacco and alcohol corporations [81, 83]. This has important implications for public health proponents working to counter interference from other harmful product industries not covered in this paper.

Findings also unveiled few but important tactical variations across industries. The alcohol and SSB industries, for example, have been “privileged with high levels of participation” within international public health organizations [20]. This differs greatly from the approach taken for tobacco industry. In fact, the Framework Convention for Tobacco Control, through article 5.3, mandates that parties to the treaty protect tobacco control from vested interests and limit interaction with the industry [84]. Moodie et al. [7] explained that the presence of such public-private partnership could, in part, be due to the fact that some non-industry actors hoped that the partnerships might result in industry-initiated change such as the reformulation of unhealthy products. Another reason could be due to product characteristics and the level of evidence available; unlike tobacco, a minority of drinkers are addicted to alcohol and the addictive properties of SSB are still being explored [85, 86]. However, given that 1) the three industries utilize similar tactics, 2) public-private partnerships at the national or international levels are not backed by strong evidence of their effectiveness [7], and 3) the health burden posed by consuming alcohol and SSB products, some have strongly argued that measures used for tobacco should be applied to these industries [85].

It is important to note that, except for tobacco, the public health community continues to disagree about ethical terms of engagement for unhealthy commodities industries [7, 40, 87]. This is concerning for several reasons. Policy change can be facilitated by cohesion and divisions limit public health movements [88, 89]. Accordingly, there is an urgent need for the public health community to generate consensus for how to limit corporate autonomy in industries that harm health.

There are fewer reviews that explored alcohol and SSB industry tactics as compared to tobacco in general. Accordingly, the array of strategies used by the alcohol and SSB industries might not have been fully captured. This is especially true in the case of SSB, where we only found 10 reviews that explored SSB industry strategies. The limited, but growing, literature in this area might also explain why developing/promoting alternative solutions, infiltrating the public health scientific community, hiding industry role in research, the use of illicit trade and smuggling, and trade framing were seen primarily in the case of tobacco and alcohol. Another explanation is that the evidence surrounding the health harms of SSB only started accumulating in recent years and, as such, some of the SSB interventions (e.g. raising taxes on SSB products) are relatively new in many countries compared to tobacco and alcohol.

Likewise, while systematic reviews on the health effects of alcohol taxes exist [90], we did not find any reviews that discussed the alcohol industry undermining tax policies as we did for tobacco and SSB industries. This is likely due to the fact that only 14 reviews explored alcohol industry strategies. Given the effectiveness of raising alcohol taxes as an intervention and the growing global attention on health taxes [91], more reviews are needed in this area to help public health advocates understand the tactics used by the industry to interfere with the adoption and implementation of alcohol tax policies globally.

Finally, our research points to several ways in which corporations seek to expand their autonomy. On the one hand, findings suggest that they take advantage of a uniquely favorable economic climate to undermine democratic processes. According to Newdick [3], “The invisible hand of neoliberalism, with its emphasis on corporate autonomy, small government, low taxation and limited social welfare, obstructs the democratic capacity to promote equality” (pg 425). We found evidence of this in tactics used to influence policy making and implementation, manipulate markets, mount legal challenges, and anticipate future scenarios. On the other hand, corporations expand autonomy in less visible ways by shaping their position in the international trade regime, through additional practices such as challenging unfavorable science, and creating a positive image. This also enables corporations to accumulate vast amounts of economic, social, and political capital, further expanding autonomy. Moreover, our findings support recent research that shows how these forces combine to individualize NCDs, limit policy prescriptions, and reinforce practices of policymaking that prioritize economic interests over health concerns [24]. It is important to note that results showed that all three industries have been expanding their reach into emerging markets as sales decline in high-income countries. Given that 85% of NCD deaths occur in LMICs [6], there is an urgent need to raise awareness of the tactics used by these industries, share lessons learned, and bolster the capacity of public health advocates in these settings to counter industry interference.

Our study has several limitations. First, to ensure that the study was manageable, we only included review articles. As such, there may be some strategies that have been missed. Second, we focused specifically on SSB industry rather than the food and beverage industry in general. To minimize this limitation, we included food and beverage industry papers that discussed SSB industry tactics specifically. Third, our findings reflect the limitations of the existing literature. Given that some types of industry involvement are behind the scenes and challenging to observe, our study only captured what was accessible. Further, we did not assess the quality of the existing literature on this topic, and, finally, only English review articles were included. Future studies are needed to address these limitations. For example, an exhaustive review of non-English articles will be important to ensure that key tactics have not been missed. Moreover, other methods such as information and communication technologies used in the area of anti-corruption (e.g. whistle blowing tools, crowd sourcing platforms) could to be explored to uncover the specific tactics used behind-the-scenes.

## Conclusions

Our study showed that the tobacco, alcohol and SSB industries utilize similar strategies to expand their corporate autonomy; this includes 1) influencing government policy making and implementation, 2) challenging unfavorable science, 3) creating a positive image, 4) manipulating markets, 5) mounting legal challenges, and 6) anticipating future scenarios. This classification can inform efforts to counter industry interference in policymaking and programming and could potentially be used to inform search terms for Freedom of Information Act requests in future research. Further, more comparative studies can also be undertaken to explore the similarities and differences across other industries (e.g. motor vehicle, coal, oil and gas, pharmaceutical). It is important to note, however, that our study only captured what was accessible in the existing literature. There may likely be other covert industry tactics.

While largely similar, we also found important differences that could, in part, be explained by the different approach used by the public health community towards these industries and the limited number of reviews that explore alcohol and SSB industry tactics. Given the escalating burden of NCDs, particularly in LMICs, there is an urgent need to develop the evidence base of alcohol and SSB industry tactics. It will also be important for future studies to explore the strategies used by public health advocates to overcome industry interference. Through a scoping review, Mialon et al. (2020) [9], for example, identified an array of mechanisms that could help address and/or manage negative corporate influence on public health. Comparative case studies could also be undertaken to explore how public health advocates in different settings overcome industry interference. Tools that help assess the level of industry interference and government’s efforts to protect public health from vested interests are also critical to increase awareness and ensure accountability.

## Supplementary Information

**Additional file 1.** Extraction Table.

## Data Availability

The datasets used and/or analysed during the current study are available from the corresponding author on reasonable request.
